# QTc interval measurement in patients with right bundle branch block: A practical method

**DOI:** 10.1111/anec.13047

**Published:** 2023-01-22

**Authors:** Abolfath Alizadeh, Mohammad Amin Shahrbaf, Mohammadrafie Khorgami, Mahboubeh Zeighami, Ala Keikhavani, Hamid Mokhtari Torshizi, Zahra Teimouri‐jervekani

**Affiliations:** ^1^ Cardiac Electrophysiology Research Center, Rajaie Cardiovascular Medical and Research Center Iran University of Medical Sciences Tehran Iran; ^2^ Faculty of Medicine Shahid Beheshti University of Medical Sciences Tehran Iran; ^3^ Department of Biomedical Engineering and Physics, School of Medicine Shahid Beheshti University of Medical Sciences Tehran Iran; ^4^ Cardiac Rehabilitation Research Center, Cardiovascular Research Institute Isfahan University of Medical Sciences Isfahan Iran

**Keywords:** corrected QT, long QT, QRS complex, QT interval, right bundle branch block

## Abstract

**Background and Aim:**

Prolonging the QT interval in the right bundle branch block (RBBB) can create challenges for electrophysiologists in estimating repolarization time and eliminating the effect of depolarization changes on QT interval. In this study, we aimed to develop a practice formula to eliminate the effect of depolarization changes on QT interval in patients with RBBB.

**Methods:**

This prospective study evaluated accidentally induced RBBB in patients undergoing electrophysiological study. Two expert electrophysiologists recorded the ECG parameters, including QRS duration, QT interval, and cycle length, in the patients. The formula was developed based on QT interval differences (with and without RBBB) and its proportion to QRS. Additionally, the Bazzet, Rautaharju, and Hodge formulas were used to evaluate QTc.

**Results:**

We evaluated 96 patients in this study. The mean QT interval without RBBB was 369.39 ± 37.38, reaching 404.22 ± 39.23 after inducing RBBB. ΔQT was calculated as 34.83 ± 17.61, and the ratio of ΔQT/QRS with RBBB was almost 23%. Our formula is: (QT_with RBBB_ − 23% × QRS). Subtraction of 25% instead of 23% seems more straightforward and practical. Our formula could also predict the QTc interval in RBBB based on the Bazzet, Rautaharju, and Hodge formulas.

**Conclusion:**

Previous formulas for QT correction were hard to apply in the clinical setting or were not specified for RBBB. Our new formula allows a rapid and practical method for QT correction in RBBB in clinical practice.

## Graphical abstract

The present study introduced a practical formula to determine the QT interval in RBBB. We evaluated 96 patients with accidentally induced RBBB during electrophysiological study. We proposed that Correcting HR with the Hodges, Bazzet, or Rautaharju formulas and subtracting 25% of QRS duration from the QT interval in RBBB seems to predict the actual QTc interval in RBBB.

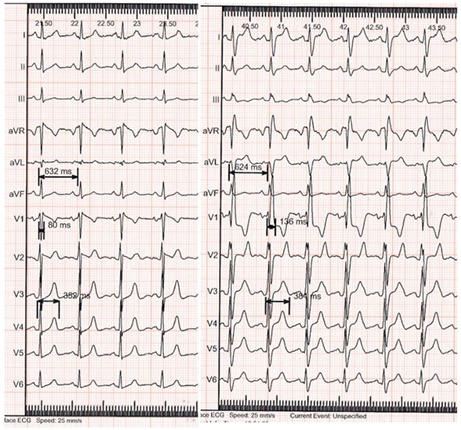

## 1 INTRODUCTION

Electrocardiogram (ECG) alterations, such as repolarization change and arrhythmia, are associated with poor outcome in patients with cardiovascular disease (CVD) (van der Bilt et al., 2009). Numerous cardiac and non‐cardiac diseases can cause alteration in ECG segments, like ionic channel pathologies and some medications that cause prolongation of QT and JT intervals (Brewer et al., 2020). Prolonged QT and JT intervals are important due to the potential risk for malignant arrhythmias and Torsade de Pointes, resulting in sudden cardiac death (Schwartz et al., 2012). In addition, the QT interval can express both depolarization disorders, indicated by QRS complex widening, and repolarization disorders, indicated by JT interval prolongation (Marafioti et al., 2018).

QT should be corrected based on R‐R interval to eliminate heart rate effects on QT interval (Yu et al., 2022). Bazett (1997) created a formula for corrected QT (QTc) calculation approximately one century ago. It is an empirical formula in standard QRS duration, but significant changes in heart rate may cause under‐ or overestimation of QTc. In addition, in patients with ventricular conduction delays, such as the bundle branch block (BBB), it is hard to precisely study the ventricular repolarization because prolonged QRS increases QT interval (Wang et al., 2017). In the context of BBB, JT interval is a better index for ventricular repolarization evaluation (Crow et al., 2003), although it is difficult to use in daily routine practice.

Some easy formulas have been developed for QT estimation in repolarization disorders (Bogossian et al., 2014; Tabatabaei et al., 2016; Yankelson et al., 2018). However, most advanced formulas are specifically for left BBB (LBBB). Furthermore, QT interval measurement in previous studies was majorly conducted through ventricular pacing‐induced BBB, and heart rate adjustment was not mentioned in most of them. There is scarce evidence for QT correction in RBBB. The present study aimed to develop a formula for estimating QT interval during intrinsic RBBB.

## 2 METHODS

### 2.1 Study design

This prospective study was conducted on patients undergoing electrophysiological study and catheterization ablation from May 2013 to March 2022 at the Rajaie Cardiovascular Medical and Research Center, Tehran, Iran. Patients were enrolled using the convenience sampling method with the inclusion criteria of age between 18 and 80 years, normal sinus rhythm (SR), lack of structural heart abnormality in the intrinsic QRS duration <120 ms, and occurrence of incidental RBBB during EP study. RBBB is defined as at least 10 consecutives wide (≥120 ms) QRS complexes with RBBB criteria. The diagnostic criteria for RBBB are wide QRS ≥120 ms, notched broad R wave in the right precordial leads, and wide, deep S wave in left precordial leads (Surawicz et al., 2009). Patients with a history of myocardial ischemia, structural heart disease, and cardiac surgery, cases with baseline RBBB in ECG evaluation, and those with a history of antiarrhythmic drug consumption were excluded.

### 2.2 Electrophysiologic study and data collection

**FIGURE 1:**
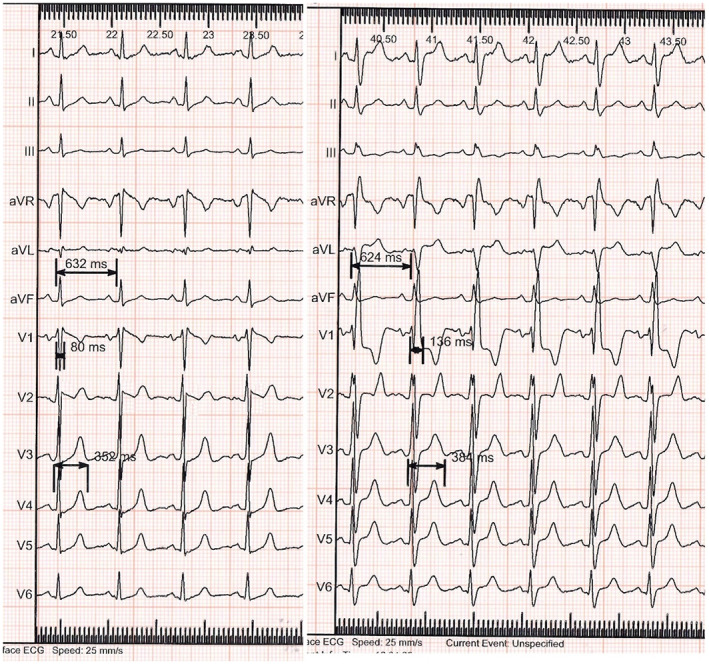
Twelve lead ECG of intrinsic narrow QRS (left side) and incidental RBBB (right side). Measurement of cycle length, QRS duration, and QT interval is shown.

All procedures were performed under conscious sedation with blood pressure monitoring and noninvasive oximetry. Standard speed (25 mm/s) and voltage (10 mm/mV) were used to measure narrow complex beats following the incidentally induced RBBB. Two expert electrophysiologists recorded the ECG parameters including cycle length, QT interval, and QRS duration as milliseconds (Figure 1).

### 2.3 Developing the formula

We redesigned the previous formula of modified QT in LBBB (Bogossian et al., 2014) to measure real QT in intrinsic RBBB. QT interval prolongation during RBBB was measured based on QT with RBBB minus QT without RBBB (ΔQT). Then, ΔQT was divided by QRS with RBBB to obtain the percentage of ΔQT based on QRS duration at RBBB (ΔQT/QRS with RBBB). To have QT interval without RBBB, we used this formula: [QT_with RBBB_ − (ΔQT/QRS_with RBBB_) × QRS]. We also applied the Hodge formula [QTc = QT + 1.75 × (HR‐60)] (Phan et al., 2015), Bazett formula [QTc = QT/√RR] (Dahlberg et al., 2021), and Rautaharju formula [QTc = QT × (120 + HR)/180] (Othong et al., 2019), to eliminate the possible HR effect on QT interval.

### 2.4 Statistical analysis

We used version 24 of the IBM statistical package for social science (SPSS) for the statistical analysis. Continuous and categorical variables are presented as mean ± standard deviation and percentages. Considering non‐normal distribution of data, Wilcoxon signed‐rank test was used to compare the differences between variables based on the normality distribution of data. For comparing corrected heart rate based on the mentioned formula, Mann‐Whitney *U* test was used. A *p* value <.05 was considered statistically significant.

### 2.5 Ethical consideration

The study was conducted under the Declaration of Helsinki. The medical ethics committee of the Rajaie Cardiovascular Medical and Research Center approved the protocol of this study (no. IR.RHC.REC.1401.023).

## 3 RESULTS

We evaluated 96 patients with a mean age of 44.74 ± 14.04 years, of whom 36 were women and 60 were men. The ECG parameters with and without RBBB are described in Table 1.

**TABLE 1:** ECG parameters before and after inducing RBBB

ECG parameters	Without RBBB	With RBBB	*p*‐Value^a^
Cycle length	721.84 ± 129.58	720.36 ± 131.84	<.0001
QRS duration	92.28 ± 8.65	143.51 ± 16.40	<.0001
QT interval	369.39 ± 37.38	404.22 ± 39.23	<.0001
QTc interval	414.39 ± 25.09	449.34 ± 26.9	<.0001

Abbreviation: RBBB, right bundle branch block.

^a^ Wilcoxon test.

ΔQT was calculated to be 34.94 ± 20.27. The ratio of ΔQT/QRS with RBBB was almost 23%. According to these measurements, a new formula was developed to allow rapid and easy correction of real QT in RBBB in practice. Our formula was:

$$\text{Real}\;{\mathrm{QT}}_{\text{RBBB}}={\mathrm{QT}}_{\text{with RBBB}}-23\%\times \mathrm{QRS}.$$

**FIGURE 2:**
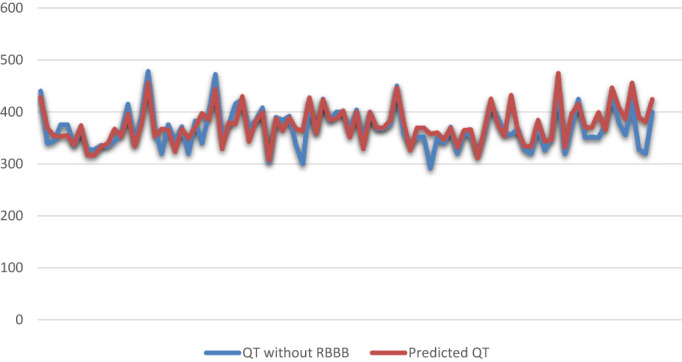
Comparing QT without RBBB with predicted QT (without HR correction)

**FIGURE 3:**
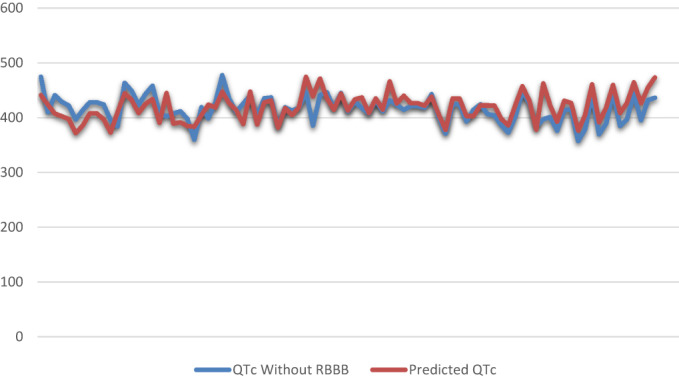
Comparing QTc interval (Hodge formula) with predicted QT

This formula can predict real QT in RBBB patients with a power of 90%. Almost 94% of the patients' QTs were between −10 and +10 percent of our formula. There were not statistically difference between the predicted QT and QT without RBBB (*p* = .26, Figure 2). Subtraction of 25% instead of 23% seems appropriate to have a more straightforward and practical formula. In order to assess the applicability of our formula, we used Hodge formula, Bazett formula and Rautaharju formula, to assess the applicability of our formula in predicting heart rate corrected QT. Our formula can predict QTc with a mean error of (1.8 ± 6.04%) for Hodge formula, (2.2 ± 7.36%) for Bazett formula, and (2.16 ± 6.98%) for Rautaharju formula. We have provided a table that suggests the QTc for each algorithm with our predicted formula (Table 2). The plot for each correction formula and predicted algorithm is presented in Figure 3. There were no significant differences between our prediction and correction formulas.

**TABLE 2:** Comparing QT and QTc based on different formula

Values	Without RBBB	Predicted	*p*‐Value^a^
QT	369.4 ± 37.38	375 ± 35.17	.26
QTc Hodge formula	414.4 ± 25.09	420.6 ± 25.15	.14
QTc Bazett formula	436.9 ± 28.25	445.1 ± 30.45	.08
QTc Rautaharju formula	419.9 ± 27.28	427.4 ± 28.19	.11

Abbreviation: RBBB, right bundle branch block.

^a^ Mann‐Whitney *U* test.

## 4 DISCUSSION

This study introduced a new formula to measure real QT interval in patients with intrinsic RBBB. The results enable electrophysiologists to eliminate QRS widening proportion to avoid overestimating QT interval and correct the heart rate variability effect on QT interval. Actual QT interval could be predicted with more than 90% power by correcting HR with the Hodges, Rautaharju and Bazzet formula and subtracting 25% of QRS duration from QT interval in RBBB. To the best of our knowledge, no previous study calculated the real QT interval during intrinsic RBBB.

The main reasons for abnormal conduction in patients with BBB are delayed depolarization and prolonged QRS duration (Rautaharju et al., 2004). JT interval interpretation was recommended in some studies due to its independence from QRS duration; however, JT interval is not commonly used in daily routine practice due to its difficult measurement and rate correction. Several formulas have been developed within the last decades to modify corrected QT in BBB (Bogossian et al., 2020). Fixed time subtraction was recommended in 1973 as QTc minus 70 ms in LBBB and minus 40 ms in RBBB (Talbot, 1973). Due to various ranges of QRS duration in patients with BBB, this formula has the risk of under‐ or overestimation of QT interval. Some other precise formulas were then developed but were not noticed in daily clinical practice as they were complex and difficult to use (Bogossian et al., 2020). In 2014, Bogossian et al. analyzed QT interval and QRS complex in narrow sinus rhythm and during right ventricular apex pacing at 10 ms below sinus rhythm. This formula suggested subtracting 48.5% of QRS LBBB from the QT interval. For easier practical use, they simplified it to QT = QT_LBBB_ − 50% of QRS_LBBB_ (Bogossian et al., 2014). Their limitation was fixed‐rate and pacing‐induced BBB. The validation of their formula and the correction of HR with the Bazzet formula (Dahlberg et al., 2021) were examined in another study on 15 patients with intermittent LBBB or post transcatheter aortic valve implantation (TAVI) LBBB. They confirmed the validity of the formula except in extreme deviations of QTc interval and/or QRS duration when the formula may miscalculate (overestimation or rarely underestimation) of actual corrected QT interval (Bogossian et al., 2017). In another study, during the left electrophysiological (EP) procedure, artificial RBBB was induced by LV pacing with a rate of 36 ± 18 higher than sinus rhythm. Modified QT for LBBB presented by Bogossian et al. was used to validate the applicability of the formula in RBBB. They demonstrated that this formula could be applied in RBBB (Erkapic et al., 2020). However, in this study, artificial RBBB was examined, which may differ in mechanism and duration from intrinsic BBB. Artificial pacing‐induced RBBB has a different configuration than intrinsic RBBB (Wu et al., 2020). It is broad and complies with bizarre morphology and abnormal components in both initial and terminal portions of QRS. Intrinsic RBBB has an initial normal QRS configuration due to normal left ventricular depolarization. However, the terminal part of QRS is abnormal due to delayed right ventricle depolarization. Besides, fixed rates for pacing at lower cycle lengths were used, and heart rate variability could not be examined. Intrinsic BBB may vary from 120 ms to more than 200 ms (Bogossian et al., 2020), but pacing induced RBBB in the study mentioned above was 175 ± 21 and 179 ± 20 in two groups of pacing compared to 143.51 ± 16.40 in the population of 96 patients in our study. Previous studies were based on artificial BBB and their validity for RBBB was checked in pacing induced BBB. Thus, our study is the first to develop a new formula to determine real QT in a higher number of patients with intrinsic RBBB compared to previous studies. Heart rate correction was done using Bazzet, Hodge, and Rautaharju formulas. While acceptable mean error was calculated for all three formulas, Hodge formula seems to be more accurate for our formula, as it was previously seen in Erkapik et al study (Wu et al., 2020).

Although, this study had some limitations. First, this study eliminated the depolarization effect on QT interval but did not evaluate other parameters influencing repolarization alteration. Second, RBBB induced with disorders, such as congenital heart disease or myocardial ischemia, was not assessed specifically. However, due to a relatively large sample of patients with intrinsic RBBB used in this study, the developed formula can be practically and accurately implemented for this group of patients.

## 5 CONCLUSION

The present study introduced a practical formula to determine the QT interval in RBBB. Correcting HR with the Bazzet, Rautaharju, and especially Hodges formula and subtracting 25% of QRS duration from QT interval in RBBB seem to predict actual QTc interval in RBBB.

## AUTHOR CONTRIBUTIONS

*Study concept and design*: Abolfath Alizadeh. *Data collection*: Abolfath Alizadeh, Mahboubeh Zeighami, Ala Keikhavani, Zahra Teimouri‐jervekani. *Analysis and interpretation of data*: Mohammad Amin Shahrbaf, Zahra Teimouri‐jervekani. *Drafting of the manuscript*: Zahra Teimouri‐jervekani, Mohammadrafie Khorgami. *Critical revision of the manuscript for important intellectual content*: Abolfath Alizadeh, Mohammad Amin Shahrbaf, Ala Keikhavani. *Approval of the final draft*: Abolfath Alizadeh, Zahra Teimouri‐jervekani, Mohammad Amin Shahrbaf.

## CONFLICT OF INTEREST

The authors have no conflicts to disclose.

## Data Availability

Data available on request from the authors.
